# Supplementary figures and images for: Layer-Specific Glutamatergic Inputs and Parvalbumin Interneurons Modulate Early Life Stress-Induced Alterations in Prefrontal Glutamate Release during Fear Conditioning in Pre-adolescent Rats
Source: eNeuro. 2025 Nov 6;12(11):ENEURO.0073-25.2025. doi: 10.1523/ENEURO.0073-25.2025 (PMC12594443; doi:10.1523/ENEURO.0073-25.2025)

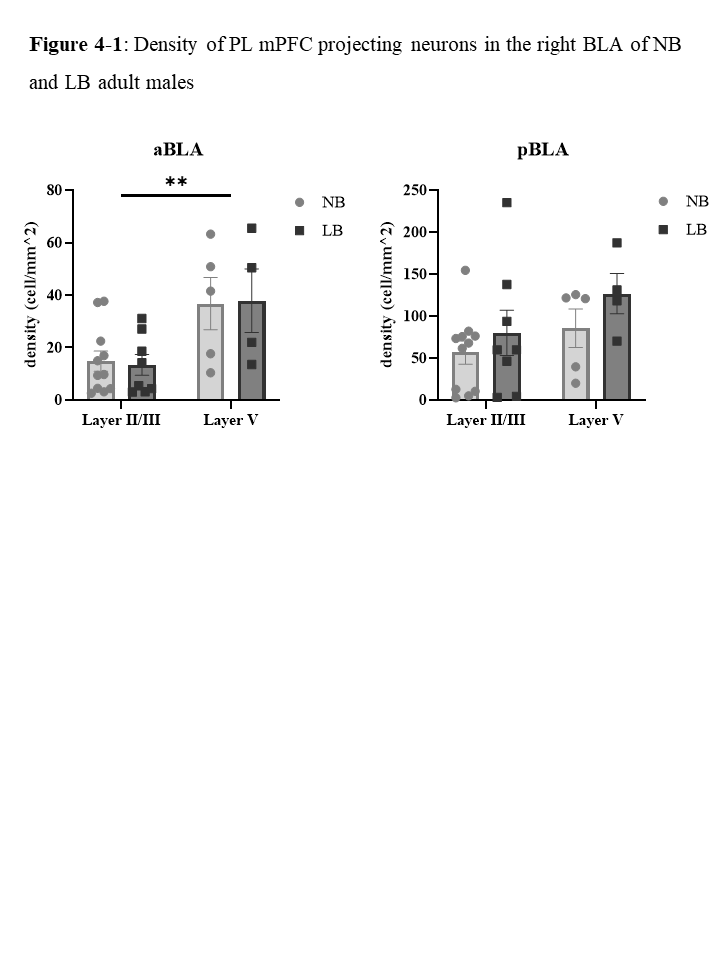

Supplement: Figure 4-1 — Download Figure 4-1, TIF file. [file eneuro-12-ENEURO.0073-25.2025-s002.tif]
